# Choking/Strangulation During Sex: Understanding and Negotiating “Safety” Among 18-35 Year Old Australians

**DOI:** 10.1007/s10508-025-03097-3

**Published:** 2025-02-05

**Authors:** Isabella Conte, Leah S. Sharman, Heather Douglas

**Affiliations:** 1 Melbourne Law School, The University of Melbourne, 185 Pelham Street, Carlton, VIC 3010 Australia; 2 School of Psychology, The University of Queensland, Brisbane, QLD Australia

**Keywords:** Strangulation, Safety, Consent, Breath play, Choking, Sexual choking

## Abstract

Despite its potentially fatal consequences, sexual choking/strangulation is an increasingly common sexual practice. Existing research suggests that its growing prevalence is partly a consequence of its popular construction as “risky yet safe.” Few studies have directly examined how people frame and engage in choking/strangulation in terms of “safety”—what people think it means for strangulation to be (or not be) “safe” and how people think safety during strangulation can be achieved. Drawing on qualitative text responses from a survey collected in 2023, this paper examined whether and how Australian adults aged between 18–35 perceived choking/strangulation in terms of “safety.” We identified and discussed four main themes arising from the data: choking/strangulation during sex can be safe; pressure/intensity is a safety mechanism; consent is a safety mechanism; and trust and communication are necessary. In consideration of these themes, we suggest that many Australian young people are not aware of the risks of choking/strangulation during sex and frequently equate pressure/intensity and consent, within a trusting relationship, with safety. Given the significant risks and harms associated with any sexual choking/strangulation and the resultant difficulty in achieving safe use, we concluded that appropriate education and information should be provided to young people.

**Supplementary Information:**

The online version contains supplementary material available at 10.1007/s10508-025-03097-3.

## Introduction

Erotic asphyxiation refers to restricting oxygen to enhance sexual pleasure, arousal, or orgasm (Herbenick et al., 2021a). Strangulation is a common method of erotic asphyxiation, involving the external compression of the neck and consequent restriction of respiration and/or blood circulation (Foley, 2015; Sharman et al., 2023). Whether performed manually (e.g., using hands, forearms or legs) or with a ligature (e.g., ties, belts or cords), strangulation is highly dangerous. Following domestic violence and assault literature, clinical symptoms associated with being choked/strangled include neck pain, chronic headaches, cognitive dysfunction, post-traumatic stress disorder and, although rare, death (Bichard et al., 2022; Sorenson et al., 2014; Zilkens et al., 2016). Given that some of the negative consequences may not develop until well after the choking/strangulation has ended, people may not connect consequences to the strangulation (De Boos, 2019).

Despite these potential negative effects, choking/strangulation has become an increasingly common feature of sex, particularly among young people (Daminato et al., 2024; Herbenick et al., 2021b, 2022a, 2023c; Sharman et al., 2024). A possible explanation for the growing prevalence of sexual choking/strangulation among young adults (Herbenick et al., 2023c) is an overconfidence in the safety of the practice. Studies indicate that sexual choking is often portrayed in pornography and media—both mainstream (e.g., Men’s and Women’s Health Magazines) and social (e.g., TikTok)—as dangerous but manageable with appropriate technique(s) and boundaries (Herbenick et al., 2023a, 2023d). To date, few published studies have examined how members of the general public frame and engage in sexual choking/strangulation in terms of “safety” (Herbenick et al., 2023c). This study aims to contribute to this emerging literature by drawing on data from a survey of Australian adults to explore whether and how adults aged 18–35 perceive “safety” as possible during sexual choking/strangulation.

### Literature Review

#### Strangulation, Choking or Sexual Choking?

The literature is currently complicated by varied terminology (Pritchard et al., 2017). In both mainstream media and social media, strangulation is commonly referred to as “choking” or “sexual choking” (Herbenick et al., 2023a, 2023d). In BDSM and kink communities, it is often described as a form of “breath play,” although this practice also includes other forms of asphyxiation (Schori et al., 2022; Wiseman, 1996). Meanwhile in medical and legal scholarship, often concerning sexual and domestic assault/homicide/femicide, it is frequently referred to as “strangulation” or “non-fatal strangulation” (Douglas & Fitzgerald, 2014; Edwards & Douglas, 2021; Glass et al., 2008; White et al., 2021). In policing, restraint of a person by the neck has been described as a “chokehold” (Gardner & Al-Shareffi, 2022).

While recognizing the context-specific nature of these various terms, we adopt the term “choking/strangulation” to reflect its common use in sex and in recognition of its medical and legal use. However, when referring to survey research that adopts any of the abovementioned terms, we adopt the language of that particular study and in the presentation of our results we maintain survey participants’ language in direct quotes.

#### The Harms and Risks of Choking/Strangulation

The health consequences of choking/strangulation are well documented. Their form and severity are reported to be contingent upon the method, intensity and frequency of choking/strangulation (Iserson, 1984; Plattner et al., 2005; Zilkens et al., 2016). These consequences range from “mild and transient” (e.g., bruising, difficulty swallowing, headaches; Zilkens et al., 2016) to alterations in consciousness (e.g., dizziness, blurred vision, unconsciousness; Herbernick et al., 2022a). Both non-consensual and consensual strangulation have been found to result in unconsciousness and show associations with mental illness including depression, anxiety disorders, suicidality and disassociation, and cognitive disfunction including memory loss and impaired concentration (Bichard et al., 2022; Busse et al., 2015; Herbenick et al., 2022b).

While death is a rare consequence of strangulation, its occurrence in both consensual and non-consensual sexual interactions is possible (Iserson, 1984; Olson, 2012; Schori et al., 2022). Indeed, sexual strangulation/choking is the most common cause of death in consensual BDSM-related fatalities (Schori et al., 2022). Similarly, non-consensual strangulation in the context of domestic violence has been identified as one of the highest markers for, and prevalent mechanisms of, femicide (Glass et al., 2008; Sorenson et al., 2014).

Most often documented injuries associated with choking/strangulation are invisible (Di Paolo et al., 2009). In a review of 300 domestic violence cases involving non-fatal strangulation in the USA, McClane et al. (2001) found that 150 victims/survivors (50%) presented no visible signs of external injury. In a more recent qualitative study involving interviews with survivors of non-fatal strangulation, Douglas and Fitzgerald (2022) found that only 25% recalled having visible physical injuries (e.g., bruising, scratch marks and finger-marks). Some participants further reported sustaining delayed injuries as a consequence of being strangled in a violent relationship, including post-traumatic stress disorder (Douglas & Fitzgerald, 2022).

These findings are consistent with other studies observing that the appearance of injury is not always immediate, but rather can be delayed. As Zilkens et al. (2016) note, clinical signs of strangulation may not appear for 24–36 h following an instance of non-fatal strangulation. For example, laryngeal edema (i.e., neck swelling) may only develop 36 h after an instance of strangulation, in rare cases resulting in delayed airway obstruction and fatal airway collapse (Di Paolo et al., 2009).

Scholarship concerning the health outcomes and consequences of choking/strangulation is primarily informed by research addressing it in domestic violence, intimate partner violence, and femicide contexts (Herbenick et al., 2023c). Therein, strangulation presents a serious risk profile; potentially different to the risk profile of choking/strangulation during sex that is typically consensual, frequent, and enacted with a range of pressures with the intention to be pleasurable, rather than cause fear or harm (Herbenick et al., 2023c). In a recent Australian survey, less than 8% of people reported that the pressure used the last time they were choked during sex was “just resting” pressure with others reporting varying levels of firmer pressure being used (Sharman et al., 2024). This is not to suggest that choking/strangulation occurring in consensual sex cannot present (or effect) serious health outcomes. Indeed, emerging research has identified that frequent and recent sexual choking/strangulation is associated with altered brain morphology and changes to neural activation patterns within working memory tasks (Hou et al., 2023; Huibregtse et al., 2022).

#### The Prevalence of Choking/Strangulation

Historically, choking/strangulation during sex was appreciated as a niche pastime (Moore & Khan, 2019); an “edgy form” of BDSM play (Douglas et al., 2024). However, recent studies on sexual choking/strangulation in Australia, the USA and Iceland identify sexual choking/strangulation as a rapidly growing trend among young adults (Daminato et al., 2024; Herbenick et al., 2023c; Sharman et al., 2024; Vilhjálmsdóttir & Forberg, 2023).

In Australia, prevalence rates from a national survey of 18–35 year olds showed that more than half of participants had previously engaged in strangulation, either having been strangled (57%) or having strangled a partner (51%) during sex, and 44% having done both (Sharman et al., 2024). Overall, results showed a gendered pattern with women more likely to have been strangled and men more likely to report having strangled partners while trans and gender diverse participants were more likely than men or women to have ever been choked or to have choked their partners (Sharman et al., 2024.) Regardless of gender, in their reported experiences, participants generally viewed their experiences of being strangled or strangling a partner positively. Similarly, in a recent probability survey of 4168 US undergraduate students (49.9% women, 48.6% men), Herbenick et al. (2023a) found that 26.5% of women, 6.6% of men and 22.3% of transgender and gender non-binary participants had been choked during their most recent sexual encounter. Additionally, 5.7% of women, 24.8% of men and 22.3% of transgender and non-binary participants reported having choked their partner during their most recent sexual event (Herbenick et al., 2023a). In interviews with 24 women about the experience of being choked during sex, experiences were often linked to positive feelings, “such as pleasure, excitement, intimacy, caring and enhanced emotional connection with partner during sex” (Herbenick et al., 2022b, p.1112).

Consistent with these findings in the USA, a BBC-commissioned national survey of 2,002 British women, found that 38% reported being “choked” during sex (Savanta: ComRes, 2019). Of this sample, 54% of women were aged 18–24, and 41% of women were aged 25–29. In a parallel national survey of 2049 British men aged between 18 and 39, 35% reported having choked someone during consensual sexual activity. Of this sample, 40% were 18–29 years old (Savanta: ComRes, 2020).

#### The Perceived Safety of Choking/Strangulation

Sexual strangulation is understood in BDSM literature as a risky and dangerous act. For example, in *SM 101: A Realistic Introduction,* Wiseman emphatically frames strangulation as inherently dangerous, with the minimization of its risks a near-impossible task: “As a person with years of medical education and experience, I know of no way whatsoever that either suffocation or strangulation can be done in a way that does not intrinsically put the recipient at risk” (Wiseman, 1996, p. 387). However, despite the well-established understanding of the risks of sexual strangulation in BDSM literature, an emerging explanation for the apparent normalization of sexual choking/strangulation—and one with which this article grapples—is the popular construction of choking/strangulation as dangerous yet safe, and manageable with appropriate techniques and boundaries (Beres et al., 2020; Douglas et al., 2024; Herbenick et al., 2023b; Sharman et al., 2024; Wright et al., 2023).

Pornography, mainstream and social media have been identified as driving this normalization. Content analyses of pornography observe the minimization, or often omission, of the risks and negative outcomes associated with sexual choking (Wright et al., 2023). Pornographic depictions of sexual choking often glorify the behavior as a hallmark of rough sex and a pleasurable and risk-free act (Moore & Khan, 2019; Wright et al., 2023). Such depictions, researchers observe, are equally present in mainstream and social media (Engle, 2020; Herbenick et al., 2023a).

On social media (e.g., TikTok, Instagram and Facebook) users commonly portray choking/strangulation (humorously) as a risky yet normal and safe feature of sex. In their analysis of 316 visual and textual memes, Herbenick et al. (2023d) found the risks associated with sexual choking to not only be minimized, but to also be romanticized. Certain memes went so far as to glorify non-consensual choking during sex (e.g., “when she says ‘choke me daddy’ and you get carried away and now she’s dead,” “when the choking went a bit too far but you’re happy she opened her eyes again”; Herbenick et al., 2023d, p. 1305).

In their analysis of 27 online articles addressing sexual choking, Herbenick et al. (2023b) found the risks associated with the behavior were minimized through often misleading references to “experts” and the provision of “safety precautions” implementable prior to, and during, the act of sexual choking rather than through humor.

With respect to safety precautions, Herbenick et al. (2023b) identified three primary mechanisms promoted among the articles: consent/communication, education, and pressure. More than three-quarters (78%) of the articles reviewed stressed the necessity of seeking consent for sexual choking, with half emphasizing the importance of verbal consent and many suggesting the additional use of “safe gestures or tap-outs” in recognition of the fact that people are not always able to speak while being choked (Herbenick et al., 2023b, p. 47). With respect to harm reduction prior to choking, 51.9% of articles suggested readers seek training or education (Herbenick et al., 2023b). The need to learn the anatomy of the neck and CPR/first aid was mentioned in 14.8% of articles. Beyond external educative sources, a few articles encouraged discussions with partners about their health (e.g., asthma, heart conditions) and trauma (Herbenick et al., 2023b). With respect to safety during sexual choking, 74.1% of articles encouraged the avoidance of the trachea/windpipe, while others advised readers to continuously “check on” their partner (e.g., observe their bodily reactions; 37%) and to limit the duration of choking (22.2%; Herbenick et al., 2023b).

Beyond these content analyses, few studies have attempted to gauge people’s perceptions of the safety of choking/strangulation and the alleged techniques used to mitigate its risks. Two qualitative research interview studies with young adults conducted by Herbenick et al., (2022b, 2022c) found most (if not all) participants perceived their own experience of choking/strangulation during sex to be safe or safer than other forms of rough sex. Commonly cited precautions that interviewees reported to take to mitigate risks included consent/communication and pressure/intensity—precautions mirroring those found in Herbenick et al.’s (2023b) content analysis of online articles. Consent assumed four different forms among the participants: verbal consent, non-verbal consent, assumed consent and non-consent (Herbenick et al., 2022b, 2022c). In terms of pressure, both men and women shared similar perceptions as to the amount and location of pressure to apply to the neck to avoid injury. Most participants believed that lower levels of pressure applied to the side of the neck, rather than the front, mitigated harm however, most if not all participants had never sought out information, education, or training on how to mitigate the risks associated with choking/strangulation (Herbenick et al., 2022b, 2022c). Despite their often neutral or positive experiences, some people may not be aware of or associate brain injury and other delayed effects with choking/strangulation (Herbenick et al., 2022b, 2022c). These precautions were either based on “common sense” or information incidentally found on social media (Herbenick et al., 2022b, 2022c). This raises serious doubts as to how informed individuals are when engaging in this risky behavior; or more specifically, how over-confident/mis-informed they might be.

This study seeks to build upon these findings by drawing upon data from qualitative textual responses to understand perceptions of the safety of choking/strangulation among Australians aged 18–35 years.

## Method

This research explored qualitative text responses from a single question taken from a larger study on sexual choking/strangulation (Sharman et al., 2024) regarding their thoughts or insights on sexual choking. The broader survey was quantitative and focused on participants’ perceptions of sexual choking, their experiences (if any) of consent and positive or negative outcomes, how safe they felt choking during sex could be, and their perception and knowledge of existing legislation around strangulation.

Toward the end of the survey as part of an experimental paradigm to be reported elsewhere, three-quarters of participants were randomly provided a few sentences with more information around possible consequences of choking during sex, quoted below:

Generally, people who have been choked/strangled show few or no signs of visible injury immediately following the event and injuries can take days to months to appear. However, it can take as little as 10 seconds for a person being strangled to become unconscious, and death can occur within 2 minutes.

Participants were then asked, “What are your thoughts or insights regarding choking during sex?” and provided a text box to respond if they wished. As the item was the last question of the survey, participants already had time to reflect on their experiences, perceptions, understanding of sexual choking/strangulation and safety before answering.

Overall, 3,209 text responses were provided from 5054 total survey participants. Due to the number of qualitative responses received, this study focuses only on those that raised the overarching concept of “safety” or “risk” related to strangulation during sex to ensure we could adequately capture the nuance of these responses focused on the research question. We note that other responses included a broad range of other concepts not related to safety and these are provided, alongside examples, in the supplementary material. However, more than one concept was frequently captured within a single participant depending on the length and diversity of their response.

### Participants

Of the survey participants who provided qualitative text responses, 1528 raised the concept of safety or risk. Demographics were collected as part of the larger survey, and those of safety/risk participants are shown in Table 1. Analysis showed no age differences among participants who provided safety/risk responses (*M*_*age*_ = 27.03 years) compared to those who provided other qualitative responses (*M*_*age*_ = 26.99 years). However, differences were observed across choking/strangulation experience. Relative to those who did not discuss safety/risk, those who did included fewer men and heterosexual individuals, and more women and bisexual participants. No differences were found for gay or lesbian participants. Compared to those who did not discuss safety/risk, participants who discussed safety/risk were more likely to report ever being sexually choked and to report having sexually choked someone else (see supplementary material for comparisons).

**Table 1:** Participant demographics and choking participation

	Total	Straight	Gay or lesbian	Bisexual	Other sexuality
	(*n* = 1528)	(*n* = 1204)	(*n* = 69)	(*n* = 178)	(*n* = 67)
Men	696 (45.5%)	605	44	34	10
Women	803 (52.6%)	599	19	136	45
Gender diverse (non-binary)	27 (1.8%)	0	6	8	12
*Had been choked* (*n* = 992)					
Men	395 (39.8%)	327	35	25	10
Women	582 (58.6%)	417	12	121	45
Gender diverse (non-binary)	15 (1.5%)	0	2	6	6
*Choked someone else* (*n* = 855)					
Men	467 (54.6%)	406	27	26	7
Women	371 (43.4%)	260	11	77	22
Gender diverse (non-binary)	17 (20.7%)	0	3	6	8
*Both choked and choked another* (*n* = 741)					
Men	368 (49.7%)	310	27	23	7
Women	359 (48.4%)	251	10	76	21
Gender diverse (non-binary)	14 (1.9%)	0	2	6	6

Transgender men and women have been included in their appropriate gender categories. Sexuality totals may not match gender totals where participants chose not to disclose their sexuality

### Analysis

All text responses were generally short, containing one to ten words (*n* = 1859, 58%), with longer responses containing two to four sentences (*n* = 1244 39% of responses). A small portion (*n* = 390, 12%) of responses were longer than ten words. All qualitative responses were thematically analyzed and were first inductively and reflexively coded. Because of the length of responses, coding and themes were approached at a semantic or surface level, rather than a deeper level of “latent” meaning (Braun & Clarke, 2006). Coding and thematic analysis were completed by the primary author with the other authors assisting to refine codes and themes across four meetings. This refinement was initially focused on the coding process and concerns with labeling and meaning. Other meetings were more reflexive in nature and assisted the primary author with rigor and insight, which included discussions of differing patterns within the data and improving the clarity of codes and themes. Although the other authors provided feedback, there was no coding reliability utilized in order to retain the reflexivity and subjectivity of the analysis by the primary author (Braun & Clarke, 2019).

The authors span three generations and have varying research backgrounds in intimate partner and family violence research, law, criminology, and psychology that impacted their positioning in the analysis. As researchers, we share a concern that strangulation consensual or otherwise is a potentially risky practice and this has likely informed our approach.

### Procedure

A stratified national sample of 18–35 year olds across Australia were collected through the Online Research Unit (ORU https://www.theoru.com), who recruited participants and distributed the survey through established research panels. Potential participants who, at panel sign up, previously identified as aged 18–35 were recruited via emails about the availability of a “new online survey”. No further information was provided to participants in the recruitment email. The embedded link then took them directly to the information sheet and consent if they wished to participate, which described the study. Response rate for the survey was 20%, likely reflective of having no information about the purpose of the study within recruitment emails. However, of those who began the survey there was an 86% completion rate. Detailed information on sampling procedures is provided elsewhere (Sharman et al., 2024).

Given that the survey was confidential and voluntary, in the results below, following verbatim quotes we provide participants’ gender, age, sexual orientation and previous engagement with strangulation during sex. For simplicity in identifying differences in engagement, we refer to people who identified they had been sexually choked through the survey as a “recipient,” those who identified they had sexually choked partners as an “actor,” those who had been a recipient and an actor as “both,” and those who reported to have never participated in either as “never engaged.” This was identified via questions in the larger survey asking participants if they had ever experienced a list of different sexual choking behaviors, which are detailed elsewhere (Sharman, et al., 2024), and were cross-checked for consistency among text responses throughout the survey.

## Results

We identified four overlapping themes related to participants’ understanding of the safety of strangulation during sex and the precautions that could make strangulation safe and reduce its harm including the perceptions that: (1) choking can be safe; (2) intensity/pressure is a critical harm reduction mechanism; (3) consent to choking is part of safety and (4) trust and communication are key. We discuss these themes below.

### Choking Can Be “Safe”

Many participants perceived choking during sex to be permissible (i.e., “fine,” “okay,” “good”) as long as it was undertaken “safely.” Here, participants implicitly conveyed a belief that safe choking during sex is possible. However, the meaning of “safely”/“safety” varied among participants. Consistent with previous research (Herbenick et al., 2022b, 2022c), most participants invoked an unexplained (yet, widely shared) notion of “safety” as a shared understanding between partners. For example: “As long as both parties are being safe about it, it’s fine” (woman, 32 years, straight, recipient).

Some participants elaborated on the concept of “safety” by explicitly addressing the inherent risks of choking: “No amount of loss of oxygen to the brain is safe and should be considered discussed and consented to before the act takes place” (man, 30 years, straight, both). However, many participants, in recognition of the dangers of choking, specified what is needed for it to be “safe.” Factors included an awareness of the risk(s) associated with strangulation, the provision (and withdrawal) of consent, open dialogue and pre-planning (i.e., the development of a safety plan, safe words, prior agreement to use a certain level of pressure):

I think as long as it’s consensual between both parties involved and there is a conversation that take place before the sex for both parties to discuss how much pressure, whether they want to restrict airflow or just blood flow, what to expect and safe words (woman, 31 years, both).

If it is done with both parties consent (that can be withdrawn at any time), a safety plan, a safe word or signal, and prior discussion of safe levels of pressure, what to do if things go wrong, etc, it is ok (woman, 30 years, straight, both).

Overall, many of the participants reported that they felt choking during sex to be potentially safe when precautions were taken. However, it is also important to note that some participants felt that people should not engage in it at all. Sometimes these participants provided a reason for this view, for example that it was “too risky,” it was “dangerous,” “people can be seriously hurt”; or, irrespective of safety, it’s “uncomfortable,” it’s “inhuman” or “wrong”. Relatedly, a small subset of participants described strangulation as inherently, and irreversibly, harmful. Reasons included the real possibility of physically harmful consequences (i.e., “death,” “permanent damage to one’s health”), the normalization of “abuse and violence” and the “negative psychological and social effects,” although these responses are not explored further here.

### Intensity and Pressure Are Critical Harm Reduction Mechanisms

Participants frequently associated the safety of choking during sex with what they referred to as the “pressure” or “intensity” of the act. Most commonly, participants perceived choking during sex to be “safe” when effected with a low level of pressure, applied to the sides of the neck (“not the airway”): “It can be done safely if the person doing the choking uses light pressure” (woman, 32 years, straight, recipient) and “It is safe to do as long as there is communication between partners and they not apply much pressure where the other person cannot breathe” (man, 25 years, both).

For some participants, a low level of pressure amounting to “resting” or “holding” one’s hand around another’s neck, or “sensual touch,” was equated with safety: “I think it should be done safely and with minimal pressure, it’s more so the action of choking that should be exciting rather than the act restricting breathing and causing discomfort” (man, 34 years, straight, both); “I think when a lot of people say choking during sex, a lot (if not most) normal people actually just mean someone’s hand wrapped around another person’s throat, with little to no pressure being applied, that’s all I’ve ever engaged in” (man, 27 years, straight, actor). Some participants indirectly suggested that moderate/“mild” intensities of strangulation, sufficient to “lightly” restrict blood flow, was “safe”:

My partner likes a firm hand on the throat but more so not choking off the windpipe, but lightly restricting the blood flow when she can feel an orgasm building up (man, 31 years, straight, both).

If it’s not done aggressively or with malicious intent, but rather just firm enough to have some pressure but not to the point where they literally can’t breathe and could sustain injuries, it can be safe (man, 25 years, gay, both)

Interestingly, in his comment above the respondent conflates intent with safety, suggesting that he may understand safety to be bound to the actor’s aims rather than to the act itself. Exactly how participants recognized pressure to be sufficient to restrict blood flow, however, was not specified. Most participants who commented on pressure perceived high levels of pressure, sufficient to restrict/impair breathing, as excessive, dangerous and consequently unsafe. As one participant stated: “[C]hoking is only okay when light pressure is placed around the neck with one hand. Almost as if you are just resting your hand there. Nothing more. Otherwise it feels violent and almost abusive” (man, 24 years, straight, both). While some acknowledged the pleasure individuals derive from high-intensity strangulation, others queried its necessity and apparent popularity:

There are so many different levels of choking. It can go from simply having your hand resting on someone’s neck, to restricting their breathing. Personally, I have never even thought about the more extreme side of choking (extreme pressure and restricted breathing) because that isn’t something my partner and i have ever done, and we probably never will. I don’t see how the extreme choking is necessary, and since it is dangerous as well as scary i’m unsure why it is so common (woman, 18 years, straight, both)

Nevertheless, participants emphasized the need for education, communication and consent with respect to the intensity (and thus the “safe” levels) with which choking during sex is exercised: “I think people should learn what amount of pressure is safe for choking” (man, 24 years, gay, never engaged) and “I think there should be a conversation before hand about how hard and how much pressure” (woman, 24 years, straight, both).

In summary, and consistent with existing research (Herbenick et al., 2023b), many of the participants in this study reported strangulation during sex to be safe when enacted with little pressure.

### Consent to Choking Is Part of Safety

Participants often tied “safety”—be it emotional or physical—to “consent” with respect to choking during sex. Certain participants recalled experiences of non-consensual strangulation—experiences they described as “scary”—emphasizing what they felt was the necessity of consent for the safe performance of strangulation: “I know that men enjoy it because they see it in porn, but i[t] feels horrible and scary and there definitely needs to be consent prior to it and there usually never is” (woman, 24 years, bisexual, recipient), and

If between two consensual adults who have discussed it prior with a safety plan in place then I do not see any harm in the act however I have been subjected to non consensual choking in a previous sexual encounter which left me angry and scared (woman, 32 years, straight, both).

In their comments, participants invoked numerous mechanisms of consent including verbal, non-verbal, enthusiastic, mutual and informed. Some participants invoked their approaches to verbal and non-verbal consent to discuss the safety of choking during sex: “Should be strictly base on consensus, be aware of your partner body language and breathing and ask them whether they want to continue the activity or not if they say no respect it and back off” (man, 32 years, straight, both).

In some cases, participants described consent as requiring mutuality. Consent was often described as a two-way agreement between parties (actor and recipient), connected to specific physical elements of the act of choking (e.g., the “amount of pressure”). For example: “As long as both parties agree to it and the amount of pressure, it can be an enjoyable experience. Consent must be given” (woman, 23 years, bisexual, both). Similarly, another respondent wrote: “[P]rovided there are discussions before and after, safe technique, safe words, and mutual consent before and during the act it could be a great way to explore sex and kink with a partner(s)” (woman, 18 years, bisexual, never engaged).

This concept of mutuality contrasted with some participants’ understanding of “informed consent” which required participants to be informed of the risks. Although, according to one participant, informed consent meant only “the person getting choked understands the risks.” Irrespective of its form, consent, where raised, was regarded by participants to be an ongoing process which could be revoked or withdrawn at any and all times: “If either party revokes consent at any point, then it needs to end. … Communication and consent is vital at all points in time, from preparing to the act itself” (non-binary, 19 years, pansexual, both).

Several participants argued that consent needed to be enthusiastic, with some having engaged in it before—“I personally love it but only if it is enthusiastically agreed upon by both parties” (woman, 20 years, bisexual, both)—and others having not: “I think its fine if it happens between two adults who enthusiastically consent” (non-binary, 31 years, gay/lesbian, never engaged).

Notably, several participants commented on the limitations of consent as a harm reduction mechanism, observing that “even with consent, it [choking/strangulation] can still cause damage.” For other participants, the limitation of consent lay in the fact that it could be (or was) often overlooked (intentionally or accidentally) by actors: “The amount of men who just initiate it without asking the woman is scary and they feel entitled to do so” (woman, 35 years, straight, recipient).

Some participants identified the potential for consent to be coerced or abused, sometimes recalling previous experiences of feeling pressured to engage in choking: “It’s very concerning and scary when it’s been done to you without your consent. It’s also sad that I can’t say no” (woman, 24 years, straight, recipient) and “[I] think a lot of men pressure their sexual partners into it” (woman, 25 years, straight, both).

Several participants explained they felt pressure to comply because of the risk of mistreatment if they attempted to withdraw their consent: “[Y]ou don’t want to say no because sometimes they’ll treat you poorly, judge you or even just do it anyway without consent” (woman, 24 years, pansexual, recipient).

While generally it was women participants who identified concerns about coerced or pressured consent regarding choking, some men stated they felt pressure from their partners to strangle them: “I get scared to do it but my partner kinda makes me feel like i have to sometimes” (man, 24 years, straight, actor). Notably one participant went so far as to reject the notion that one could consent to choking given its inherent dangers:

Personally I hate the idea and thinking about it makes me feel entirely uncomfortable. I find it a difficult issue to consider in a “consensual” situation simply because for me, it could never be consensual, and also because of the sheer risk involved to human life (woman, 31 years, straight, never engaged).

While many emphasized the necessity of consent to make strangulation during sex safe, other participants highlighted the limitations of consent as a harm reduction technique. For these participants, consent was viewed as being vulnerable to coercion and or often disregarded. The perceived role of consent in safety was also strongly linked to concepts of trust and communication which we discuss below.

### Trust and Communication Are Key

Participants’ perceptions of strangulation during sex as “safe” often circled around the importance of trust and communication: “To be done safely there needs to be a high level of trust between the participating people, and definitely consent” (man, 29 years, straight, actor). According to some participants, the safety and enjoyment of strangulation during sex is “truly based on trust”; specifically, confidence in the parties involved to be respectful and conscious of boundaries and prior agreements (concerning intensity, duration etc.):

I think if it’s something both parties would like to do then why not. The act of being choked can be an enjoyable experience as long as it stays within a certain range and you trust the other person knows your body language and cues to communicate when it’s time to stop (woman, 32 years, bisexual, both).

For many participants, the requirement of trust subsequently means “strangers,” “one-night stands” or “new partners” are not eligible parties with whom to perform strangulation during sex. For example: “If you trust your partner then choking is more exciting during sex, and if you with stranger then choking could be dangerous” (woman, 25 years, straight, recipient and actor); “I only ever engage in this with a long term boyfriend” (woman, 30 years, straight, recipient and actor) and “[i]t’s all about trust in your partner. I don’t believe I would be doing this with a complete stranger” (woman, 33 years, straight, recipient).

Participants emphasized that trust is “built up over time.” It is not an automatic certainty, especially when undertaking a risky activity like choking during sex:

I have only engaged in choking with one sexual partner, because I trust him and we have an understanding that a little pain is something we both enjoy. However, if I was to have a new sexual partner who decided to choke me the first time.. I would not be comfortable. It takes time, for me, to get to a place of trust and comfort (woman, 32 years, straight, both).

I think if it is done safety and both people consent to it then it is okay, especially in a trusting relationship or a sexual partner who you have been with for a while. This means you’ve gained trust and you can trust them when choking you. Personally I am in a 2 year relationship with someone and I trust him to be able to choke me safety without any hard pressure or feeling scared (woman, 20 years, straight, both).

However, there was a contrasting view on strangulation during sex as potentially not respectable or too risky to perform with someone you loved: “If it was a hooker hell yeah. But someone I loved I wouldn't do it” (man, 33 years, straight, both). Typically, though, participants felt that alongside a “trusted partner,” participants commented on “communication” as “key” to making strangulation during sex “safe for both people involved.” Some participants extended the scope of communication beyond consent to include the method of strangulation (i.e., ligatures, hands), the intensity and the boundaries of exploration. A few participants identified certain systems of communication, such as the “traffic light and tap out system” to be effective in mitigating risks (Dunkley & Brotto, 2020). However, most participants simply regarded open, constant and clear communication as sufficient to ensure the safety and enjoyment of strangulation during sex: “[I] think if you do it safely and communicate constantly with your partner it can help enhance your sexual experience together” (man, 26 years, straight, both); “[W]ith lots of communication and consent these things can be explored safely and without consequence” (woman, 34 years, bisexual, both) and

There is a fine line between a firm hold of a neck and a violent choke. I think it all depends on if you have a long term partner that you can trust who isn’t wanting to harm you and being educated on these details such as where to hold, how much pressure, not holding for 10+ seconds and also respecting each others consent before and during (woman, 26 years, straight, recipient).

Overall, many of the participants connected ideas of light pressure, consent, communication and trust as key to ensuring the safety of choking during sex.

## Discussion

These findings underscore a complex set of perceptions about the potential for the safe use of choking/strangulation during sex. Consistent with prior research conducted in the USA (Herbenick et al., 2022b, c), most participants in our Australian survey who provided qualitative responses reported that choking/strangulation during sex could be “safe” if undertaken with what they viewed as the proper precautions. These participants generally shared a view about the approaches or safety mechanisms that they felt could be used to improve safety. Primarily participants highlighted regulating pressure as a key safety mechanism and suggested that consent, communication, and trust were integral to safety. In contrast, other participants considered strangulation to be inherently unsafe because of the risks of physical harm and the possible psychological effects.

Participants associated the safety of choking/strangulation during sex with the level of pressure applied. While a number of participants said that hands “resting” or “lightly” touching the neck ensured the safety of choking/strangulation, other participants suggested that “light” restriction of the neck was safe. Plattner et al. (2005) observed that “it would be wrong to consider any grasp of the neck as life threatening” (p. 204) and that increasing intensity and duration of pressure increases the dangers of strangulation. Some participants were clear about using reduced pressure with reference to obstructing airflow, but it was unclear if those who placed pressure on the neck targeting blood flow were doing so because they felt it was less risky, and whether they felt or believed they could safely use more pressure on this part of the neck. Research shows there is a “fine line” (of relatively low pressure) between fatal and non-fatal strangulation (Shields & Hunsaker, 2020) and sexual choking/strangulation is the leading cause of death in BDSM play (Schori et al., 2022). It may be that many people who use choking/strangulation during sex are using safety strategies that do avoid serious negative outcomes, they may simply be lucky, or they may be unaware of the harms caused to them by the behavior. Given the relatively low level of pressure needed to cause death from choking/strangulation even a light restriction of blood or airflow could have negative implications for a person’s health. For example, researchers have emphasized the short time within which unconsciousness can occur, as between 8 and 18 s (Sauvageau et al., 2011). Increasingly research has shown the association between unconsciousness and brain injury. For example, in studies with women who have experienced choking/strangulation during sex, even where they did not lose consciousness, compared with women who have not, differences in brain structure were identified (Hou et al., 2023) and differences in working memory were observed (Huibregtse et al., 2022) between groups. While these changes may not be noticed initially by affected individuals, when choking/strangulation is repeated, the injury to the brain, and associated negative effects on working memory, cumulate (Huibregtse et al., 2022). In the context of sex, where alcohol and other drugs may be used, the difference between resting and light pressure may be difficult to discern exposing the person being choked/strangled to increased risk of ongoing injury. Notably BDSM practitioners reject the use of drugs and alcohol during BDSM plays as it makes it unsafe and is considered irresponsible (Schori et al., 2022).

Similar to other studies (Herbenick et al., 2022b, 2022c), many participants in the Australian survey emphasized that consent was pivotal to ensuring strangulation during sex was safe, but whether and how consent was negotiated varied widely. In general, participants associated consent with mutuality and understood it as an ongoing process where a person could withdraw when they wished to. On this latter issue we note that one study has found that a person being strangled may not be able to withdraw their consent by gestures or words, despite wanting to (Rossen et al., 1943). Some participants observed that consent should be informed consent, that is that participants should be informed of the risks associated with choking/strangulation in order to provide true consent. However, it is clear from many of the survey responses that the risks and dangers associated with choking/strangulation were not appreciated by all participants, even when they identified the need for informed consent. This is of concern given the literature indicating that for most young adults, pornography, mainstream and social media are the primary sources of information (Herbenick et al., 2023d; Sharman et al., 2024), none of which have been very reliable sources of information about the risks and dangers associated with sexual choking/strangulation. Furthermore, while participants identified the importance of consent in their open-ended responses and that the consent should be of a high standard (e.g., mutual, ongoing, possibly informed), women’s identified experiences of choking/strangulation within this sample rarely involved the provision of explicit verbal consent prior to being choked (Herbenick et al., 2022b, 2022c). Many women reported that a much lower standard of consent was relied on—body language or facial expressions or previous ongoing consent (Herbenick et al., 2022b, 2022c). Ultimately though, participants’ views about the role of consent as a safety mechanism were mixed with some seeing limitations to consent as it could be coerced and pressured, and this type of consent was not viewed as true or reliable consent (Harris, 2018).

Trust and communication were important themes identified in the participants’ comments about safety (Herbenick et al., 2022b, 2022c; Sharman et al., 2024). For many, to be safe sexual choking/strangulation needed to be carried out in a trusting relationship where partners could communicate with each other. Presumably this suggests that choking/strangulation is not an activity most participants associate with casual sexual experiences. This is perhaps not surprising given that strangulation is understood as a potentially risky and dangerous activity whose risks and dangers can be mitigated by trust and communication.

### Conclusion

Overall, these findings demonstrate how young adults in Australia are grappling with issues of safety regarding choking/strangulation during sex. For some, a misunderstanding of the potential dangers was evident, others bemoaned a general lack of knowledge among those who engaged in choking/strangulation. The findings underscore the importance of education and training. In some cases, participants themselves expressed a desire for greater information regarding choking/strangulation during sex because they felt they lacked knowledge about potential dangers. Others expressed a more universal need for all to increase their understanding of the risks of choking/strangulation and the need for informed consent before engaging in it. Given the private nature of choking/strangulation during sex, and the role of mainstream and social media and online pornography in influencing young adults’ sexual behaviors and attitudes toward sex (e.g., Chua et al., 2023; Dolev-Cohen et al., 2024), there is a real concern about how and to whom it is best to disseminate knowledge. A recent literature review published by an Australian non-government organization (“NGO”) recommends that women and their sexual partners should be provided with “accurate and up-to-date information about the risks associated with choking and being choked by their sexual partners” (Women’s Health NSW, 2024, p. 23). This NGO also recommends that resources should make “the clear link between choking and mild brain injuries, including the long-term effects of repeated brain injuries” (p. 24). How those resources are developed and how they should reach their target audiences is a matter for appropriate agencies who are skilled at developing and disseminating sexual health campaigns (e.g., It’s time we talked: www.itstimewetalked.com.au).

## Supplementary Information

Below is the link to the electronic supplementary material.

Supplementary file1 (DOCX 28 kb)

## Data Availability

Readers interested in the data for the current study are invited to contact the corresponding author.
