# Supplementary material for: Choking/Strangulation During Sex: Understanding and Negotiating “Safety” Among 18-35 Year Old Australians
Source: Arch Sex Behav. 2025 Feb 5;54(2):483–94. doi: 10.1007/s10508-025-03097-3 (PMC11836099; doi:10.1007/s10508-025-03097-3)
Supplement: Supplementary file 1 — Supplementary file1 (DOCX 28 kb) [file 10508_2025_3097_MOESM1_ESM.docx]

Table S1. Other coded categories not separately analysed

| **Other coded categories** | **Description** | **Quote example** | **Responses** |
| --- | --- | --- | --- |
| Blank | No response provided |  | 1845 |
| Other response | illegible, unfinished, or irrelevant to choking | “Thank you. I love sex”  “Yes” | 99 |
| Survey related response | Addresses form/structure/substance of survey | “Good survey, very insightful”  “Very weird survey” | 140 |
| Choking intentions or understanding following the survey | Reflection on information provided at the end of the survey | “I love it and intend to continue”  “I will take it more seriously when my partner choke[s] me longer after I've tapped out” | 42 |
| Ambivalent/unsure | Unsure about choking or how to respond | “It is dangerous and I’m unsure about it” | 60 |
| Choking as absolutely impermissible | Thoughts that no-one should engage in choking during sex | “I think its shit and not enjoyable and I don't think people should do it I think it’s dangerous and just not how you should go about touching a human or having sex I don't find it appealing” | 155 |
| Personal dislike/avoidance | Personally not enjoyable to respondent | “Not at all interested in it. I do understand that some enjoy it, but it's certainly not for me” | 459 |
| Past regret | Negative previous experiences or regret | “Personally, I had a terrible experience, and I will never be doing it again” | 3 |
| Choking as permissible for others | Viewing choking as acceptable | “Each to [their] own do what makes you happy” | 189 |
| Openness to partake in future | Potential for participating in choking in the future depending on the circumstances | “If my partner asked me to do it, I would consider it.” | 51 |
| Fear or concern around choking | Feelings of fear toward participating in choking during sex | “Makes me a bit fearful due to past experiences/ stories from friends” | 94 |
| Choking as being a positive experience | Choking as enjoyable and sexually stimulating | “My personal thoughts of choking is that it is fun and adds more heat in the bedroom” | 669 |
| Choking as abnormal or unnecessary | Choking as strange, weird, disgusting, unhealthy | “It is a tiny bit strange but whatever floats your boat I guess” | 390 |
| Choking as (normalising) violence | Viewing choking as violence or as normalising violence | “I think it’s a door to more violence in the relationship. There are plenty of other things to try other than something so violent.” | 47 |
| Choking as an expectation | Choking as expected during sex and normalised via porn and social media | “Pornography has greatly normalised the idea that choking during sex is a common desire rather than a kink.” | 78 |
| Choking as BDSM/kink | Choking as a ‘kink’ and descriptions of BDSM practice | “For a lot of people who play with D/s dynamics, breath play and choking is a CONSENSUAL kink. Within the scene it is done in a safe manner with aftercare always. I think it is dangerous when squares (Vanilla) people try and engage in certain kinks or fetishes that they've seen once through media and are not knowledgeable in” | 48 |
| Perceptions of criminalisation around choking | Discussion of government reach, potential criminalisation and its consequences | “I feel that consented choking should not be shamed or made illegal in any way, however, there should be education campaigns around the dangers and warning signs associated with the activity.” | 95 |

nuance

Table S2. Comparison of characteristics of safety and non-safety respondents

|  | Safety responses (n = 1528) | Other responses (n = 1681) | Significance |
| --- | --- | --- | --- |
| Age (years) | 27.03 | 26.99 | *t* (3207) = 1.72, *p* = .085 |
| Gender |  |  |  |
| Men* | 696 (45.5%) | 881 (52.4%) | $\chi$2 (n = 3171, 1) = 12.39, *p* <.001 |
| Women* | 803 (52.6%) | 791 (47.1%) |  |
| Gender diverse (non-binary) | 27 (1.8%) | 11 (0.7%) | N/A |
| Sexuality |  |  |  |
| Straight* | 1204 (78.8%) | 1420 (84.5%) | $\chi$2 (n = 3088, 2) = 9.58, *p* = .008 |
| Gay/Lesbian | 69 (4.5%) | 70 (4.2%) |  |
| Bisexual* | 178 (11.6%) | 147 (8.7%) |  |
| Other | 67 (4.4%) | 37 (2.2%) | N/A |
| Had been choked* | 992 (65%) | 810 (48.1%) | $\chi$2 (n = 3209, 1) = 92.60, *p* <.001 |
| Had choked someone else* | 855 (53.9%) | 732 (43.5%) | $\chi$2 (n = 3209, 1) = 50.31, *p* <.001 |

Note: * indicates difference between safety responses and other responses for a category
